# Commentary: Why sprint interval training is inappropriate for a largely sedentary population

**DOI:** 10.3389/fpsyg.2015.01999

**Published:** 2016-01-07

**Authors:** Mary E. Jung, Jonathan P. Little, Alan M. Batterham

**Affiliations:** ^1^Faculty of Health and Social Development, School of Health and Exercise Sciences, University of British ColumbiaKelowna, BC, Canada; ^2^School of Health and Social Care, Health and Social Care Institute, Teesside UniversityMiddlesbrough, UK

**Keywords:** high-intensity interval training, exercise adherence, affect, self-efficacy, exercise intensity, relative intensity

Herein we present a commentary on Hardcastle et al. (2014) claim that interval training is “unlikely to be taken up by the majority of the sedentary population” (pg. 1).

## Is dual mode model (DMM) applicable to interval training?

DMM posits that exercise well above ventilatory threshold (VT) elicits a negative affective response as compared to exercise below VT (Ekkekakis et al., 2011). Given work periods of interval training are spent above VT, DMM has been extended to suggest interval training will be perceived as aversive. This model has accumulated support from studies examining affective responses to *continuous* exercise in a laboratory. While this seminal work has advanced knowledge of the relationship between exercise intensity and affect for *continuous controlled exercise*, it is premature to discount interval training on the basis of this model. Interval training also involves rest/recovery periods well below VT. It is presumptuous to assume that intervals performed in an intermittent fashion will mimic experiences felt during continuous exercise. Recent research has demonstrated that varying the length of intervals impacts affect, perceptions of effort, and enjoyment (Martinez et al., 2015). With the countless permutations of intervals possible, it is conceivable that pleasurable experiences of interval training exist.

## Do improvements in affect result in greater exercise adherence?

Alongside DMM is the proposition, based on hedonic principles, that in-task affective responses predict future exercise adherence. The argument against interval training is thus: if it leads to negative affect it could never possibly be adhered to. Before the research community advocates or shuns any particular type of activity, it is imperative to examine the evidence behind the proposed relationship between affect and exercise adherence. To date, we are unaware of any randomized studies where changes in affect were induced and subsequent exercise adherence assessed. Two publications are oft-cited, wherein individuals were not prescribed exercise that elicited low or high affect, but rather affect was measured throughout an isolated treadmill test in the laboratory during a test to exhaustion (Williams et al., 2008) or during a 10-min walk test (Williams et al., 2012) and retrospective accounts of exercise behavior (not objective measures) were recorded 6 or 12 months later. It is important to note, when using these references to suggest adherence is influenced by affect, that both studies instructed participants to adhere to moderate-intensity exercise only. Data examining the relationship between affect and adherence to vigorous-intensity exercise should be used if assessing whether DMM is applicable to interval training. The goal is not to critique past work, but rather to stimulate empirical testing of whether changes in affect do indeed lead to changes in exercise adherence. Without such evidence, this suggested relationship remains speculative.

## Does interval training lead to perceived incompetence?

It was argued that interval training “is likely to be considered too arduous and may evoke anticipated perceived incompetence, lower self-esteem, and potential failure.” This statement lacks scientific evidence. Theoretically, task self-efficacy will increase when an individual succeeds in performing the specific task in question. As with *any* exercise prescription, most published interval training protocols logically require participants to gradually increase the number of intervals performed. For example, in Jung (Jung et al., 2015) and Robinson (Robinson et al., 2015) this was purposefully done to ensure that participants experienced sensations of accomplishment and mastery with interval training, and this is supported by increases in participants' self-efficacy to engage specifically in interval training. Whether such increases in self-efficacy to engage in interval training are related to reported preference and enjoyment over other types of exercise (Jung et al., 2014) is worthy of future investigation.

We agree, based on substantive literature, that exercise self-efficacy is positively related to future exercise behavior. What we caution against is claiming interval training leads to decreases in self-efficacy without testing this hypothesis, conflating the terms competence, self-esteem, and self-efficacy, and making conclusions about how interval training influences these distinct variables without solid evidence.

## How hard is interval training?

Opposition to interval training (Hardcastle et al., 2014; Biddle and Batterham, 2015) has narrowly operationalized what this type of exercise may look like. What is consistently left out is the *relative exercise intensity* innate in all prescriptions of “vigorous” exercise. Interval training is most broadly defined as brief bursts of high-intensity exercise interspersed with low-intensity recovery/rest. What is considered high-intensity for an individual who has not exercised in years looks drastically different than conjured-up notions of all-out exercise performed by athletes and in bootcamp classes. In our collective research, we have introduced interval training to undergraduate students; middle-aged sedentary, overweight, and obese men and women; individuals with prediabetes and type 2 diabetes; adolescent boys and girls; and abdominal aortic aneurysm patients. For these individuals, the relative intensity of the high-intensity exercise periods often equates to walking on a treadmill at a speed of ~3.0–3.5 mph, at a ~3–5% incline (~5–6 METS). What is particularly noteworthy is that, in individuals who have been inactive for several years, and for those who are obese, engaging in traditionally prescribed moderate-intensity continuous exercise at 3–6 METS is either not possible or extremely difficult.

## Less sterile name-calling, more interdisciplinary collaboration?

For whatever reason, there appears to be disciplinary rivalry within sport and exercise sciences on the topic of interval training. This rivalry shines through in academic writing—interval training is rarely mentioned in health and exercise psychology papers without emotional reference: “Advocating high-intensity exercise as a public health strategy – such as ‘interval training’ protocols **currently in vogue with physiologists** – is likely to fail due to low rates of adherence. **This is probably why exercise has been used as a punishment in the past**…” (Biddle et al., 2015) P.134 [bold added]. The reality is that interval training remains popular in the general public. This popularity, combined with lack of currently available long-term adherence trials and dearth of evidence on intervention-induced changes in affect and objective adherence measurements, necessitates further scientific examination. We encourage that, rather than disputing without all necessary data, physiology- and psychology-related field experts engage in interdisciplinary research for a fair evaluation of this modality.

## Author contributions

All authors contributed equally to the development of ideas and writing of this manuscript.

## Funding

MJ is supported by a Michael Smith Foundation for Health Research (Award #5917) and a Canadian Institutes of Health Research Foundation Scheme (#333266). JL is supported by a Canadian Institutes of Health Research New Investigator Award (201412MSH- 141980).

### Conflict of interest statement

The authors declare that the research was conducted in the absence of any commercial or financial relationships that could be construed as a potential conflict of interest. The reviewer Johnny Padulo and handling Editor declared their shared affiliation, and the handling Editor states that the process nevertheless met the standards of a fair and objective review.
